# A Two-Stage SEM—Artificial Neural Network Analysis of the Rewards Effects on Self Perceived Performance in Healthcare

**DOI:** 10.3390/ijerph182312387

**Published:** 2021-11-25

**Authors:** Claudiu George Bocean, Cristina Claudia Rotea, Anca Antoaneta Vărzaru, Andra-Nicoleta Ploscaru, Cătălin-Ștefan Rotea

**Affiliations:** 1 Department of Management, Marketing and Business Administration, University of Craiova, 200585 Craiova, Romania; 2 Faculty of Mechanics, University of Craiova, 200585 Craiova, Romania; cristina.rotea@edu.ucv.ro; 3 Department of Economics, Accounting and International Business, University of Craiova, 200585 Craiova, Romania; 4 Doctoral School in Economic Sciences, University of Craiova, 200585 Craiova, Romania; ploscaru.andra.k6j@student.ucv.ro (A.-N.P.); roteacatalinstefan@gmail.com (C.-Ș.R.)

**Keywords:** rewards, performance, structural equation modeling, financial motivation, recognition, equity, healthcare

## Abstract

Healthcare managers consider the rewards and performances of employees as central elements of their activities due to the challenges caused by the phenomenon of healthcare employees’ emigrating to higher-income countries, which has reduced patient satisfaction and led to a negative image of hospitals. In this context, this paper analyzes how employee rewards influence the employees’ self-perceived performances in the hospital units of the emergency medical system in Romania. Using structural equation modeling, we analyzed the relationships between the investigated variables, showing that financial motivation and the recognition of employees’ merits are central to employees’ self-perceived performances. Ensuring equity also has a positive impact on how the reward package is established and managed. While financial rewards are the most important incentives to increase efforts to exhibit higher performances, recognition has a long-term motivational effect.

## 1. Introduction

Rewards are the core drivers for any organization with highly qualified human resources. However, in healthcare, especially in emergent countries, retaining and motivating talented employees is crucial for health service quality and improving hospitals’ patient satisfaction and image, especially in public health systems [1].

The number of jobs available in healthcare continues to grow, while the total number of healthcare workers has decreased. Finding suitable rewards is an ongoing issue for organizational leaders, particularly in the healthcare field. Properly rewarded employees will perform better. Employee rewards are the essential enhancer of human resources performance; human resources ensure an organization’s performance, regardless of whether it is of the public, private or non-governmental type [2,3,4,5,6,7,8,9,10,11]. Therefore, it is necessary to assess the impact of optimal rewards on performance, especially in healthcare. Healthcare is a special field due to the challenges posed by the emigration of employees phenomenon. Therefore, identifying policies and ways to retain and motivate talented human resources is essential to provide quality medical services, increase patient satisfaction, and improve the precarious image of healthcare units, especially those in the public medical system. The concern regarding the employees’ reward, which requires important essential financial resources, is a primary concern for hospital managers in Romania [4].

The general problem faced by the Romanian medical system is that inadequate employees’ motivation has a negative impact on employees’ self-perceived performances [2]. The specific problem of the Romanian medical system, as is the case in all medical systems in emerging countries, is that some managers of hospitals do not have strategies to reward employees, which adds to the relatively poor financial resources for influencing employees’ self-perceived performances [3]. Although the financial component of the rewards has improved significantly in the last three years (the net incomes of doctors and nurses have tripled), the migration phenomenon amid employee dissatisfaction remains high. Due to the emigration of doctors to Western European countries, the poor endowment of hospitals, and poor working conditions, research is needed on the effects of improved rewards on increasing the efforts of health workers to achieve better performances.

There is a research gap in terms of how healthcare organizations can influence employees’ self-perceived performances in the absence of substantial financial resources allocated for employee compensation. The paper’s originality derives from the proposal of a multidimensional model (based on partial least squares structural equation modeling) for measuring the influence of employees’ rewards on employees’ self-perceived performances, linking the way in which reward packages are structured and managed, on the one hand, and the importance of recognition equity to Romanian employees. Based on the research gap identified, this paper aims to analyze and evaluate the components of employees’ rewards as a central element of the motivational system that influences the employees’ self-perceived performances in Romanian healthcare.

## 2. Literature Review and Hypotheses

### 2.1. Concerns about the Human Resource in the Health System

A significant problem facing the medical sector in emergent countries is the lack of specific categories of appropriate human resources to provide quality healthcare. As a result, healthcare is under severe pressure due to insufficient resources, such as doctors in certain specialties and, in general, talented medical professionals [5,12]. In addition, public hospitals have problems, such as high turnover rates, due to insufficient compensation in such public institutions [5,13,14]. In Romania, this issue has been addressed very recently by repeated wage increases and placing doctors and nurses in higher positions in the salary scale of staff in the budget sector. Furthermore, due to the emigration of doctors and nurses, fewer healthcare workers will have to manage an increasing number of patients. Therefore, better working time management is also needed in addition to financial rewards, which does not affect healthcare employees’ work capacity and performances in medical services. Another tool for an efficient reward system is non-financial reward systems and ensuring equity in the structuring and managing of the reward package [11,14,15].

Few organizational managers consider an organization’s human resources to be its main asset for achieving its proposed objectives. Organizations act and rely on different strategies to compete in the market and enhance performance [5,16], and efficient human resources are crucial to gain a competitive advantage and for their image [5,10]. Urosevic and Milijic [17] show that talented employees are essential for achieving goals and establishing an optimal organizational climate if an organization wants to achieve its goals; the authors of [5,18,19,20] showed that talented employees positively affect an organization’s results and service quality.

According to [21,22,23], human resources are even more important in the medical field because almost all employees contact the beneficiaries of health services regardless of their positions. However, the literature [10,18,19,20] has shown that although most hospitals recognize the worth of employees at the basic service level (in direct contact with the patient), some healthcare organizations do not understand the factors that motivate these employees. As a result, they either lose them, or they record lower levels of performance.

### 2.2. Factors That Affect Employee Motivation and Reward

Motivation is a process exercised by managers oriented towards obtaining results, with employees as the subjects [24,25,26,27]. The motivational process depends on many factors and the psychological characteristics of employees [28,29,30,31,32]. An individual’s reasons for working better are needs, desires, and expectations [33]. Motivation varies for each individual depending on needs, objectives, and motivational factors [24,34]. The reward system, which includes financial and non-financial rewards, is a central point of the motivational process [35].

The most widely used reward that remains a highly effective motivating factor is the financial reward [26]. However, the preponderance of a motivational tool may lead to the motivation of a specific part of the employees, while some employees become deeply demotivated [24].

Chaudhry et al. [36] highlight the evolution of public institutions in implementing group rewards and periodic additional reward distribution schemes (quarterly and annual bonuses and incentives). Payment systems that aim to stimulate performance reward people for achieving their set goals, and the payment of the compensation is proportional to the contribution of the performance. Fallon and McConnell [15] argued for the need for financial stimuli, but these are not sufficient to determine the retention of health workers, especially at medical facilities in rural or economically disadvantaged areas, as well as non-financial rewards such as providing housing facilities, recognizing healthcare professionals with better performances and providing access to additional training opportunities.

A significant factor for organizations with exponential growth, especially during economic boom periods, is non-financial incentives. Adequate rewards can keep the right employee within organizations, but additional incentives encourage workers to overcome their expected performances. Other non-financial rewards include improved work conditions, flexibility in employment, and balancing one’s private life with their job [37]. Based on the theoretical considerations, we formulated the second hypothesis of the research regarding the influence of the reward on the human resource performances in healthcare:

**Hypothesis** **1** **(H1).** *Financial motivation exerts a more significant influence than non-financial motivation (recognition) on the self-perceived performance of human resources, especially among the category of non-productive auxiliary staff (administrative and maintenance staff) and directly productive staff (nurses, laboratory staff, pharmacists), whose salaries have not increased as much as doctors’ salaries*.

A motivational theory has also been formulated concerning fair policy in the field of reward: equity theory. The distribution of rewards within organizations has essential behavioral consequences. For example, employees are rarely passive about what is happening around them at work. On the contrary, they observe events and assess them through the prism of their interests. The theory of equity is based on two assumptions: people view their situations according to the problems of other organization members in similar positions; people want equity in relationships in organizations, the information on equity being galled from the observations made [38].

Within a bilateral relationship, the two main components are inputs and outputs. The inputs are what brings an individual into a relationship, and outcomes are what results from the relationship. Equity occurs when a person’s equity rate is equal to another person’s equity rate. Therefore, the equity rate shall be expressed as the ratio between inputs and outputs [39]. The theory of equity considers that individuals aim for the yield of righteousness in their case to exceed the work of equity of others. When rates are not equal, inequity occurs, which leads to unintended consequences: reduced productivity, quality reduction, absenteeism, resignations, thefts, and sabotage. Thus, if individuals find that outputs are not commensurate with inputs, they seek to reduce their inputs to balance equity rates.

In addition, starting from the equity theory, [40] highlights the need for employees to receive fair compensation to other employees. Therefore, the employee is expected to compare his performance–reward ratio with the same ratio obtained by others [26]. Furthermore, in his study, the author of [40] notes that some supervisors in evaluating subordinates tend to over-evaluate them, affecting the correctness of a reward management system and negatively influencing performance. Therefore, perceived equity is a determining factor in motivating performance [9]. Evan and Simmons [41] pointed out that underpaid employees do not perform adequately due to perceived inequity, while overpaid employees also do not perform adequately. Therefore, they considered that the lack of equity was an essential factor in terms of financial motivation. Bao and Wu [42] focus on the perceived equity deficit, showing that inequity is perceived at the employee level more intensely in terms of non-financial motivation, while Ittner et al. [43] show that equity has an important impact on the intensity of financial motivation in the case of senior executives, lower-level managers, and non-exempt employees of “new economy” firms.

Another issue regarding equity and fairness occurs in public health institutions in Romania, where the salary scale is set centrally and does not allow sufficient flexibility. If this pay scale, a central component of reward systems, is designed without regard to the principles of fairness and equity, it will create a state of dissatisfaction and frustration.

Based on the theoretical considerations regarding the importance of equity theory in managing employees’ performances, we formulated the second hypothesis of the research on the influence of reward on the performance of human resources in healthcare:

**Hypothesis** **2** **(H2).** *Ensuring equity positively impacts the reward package establishment and management*.

The differentiated reward is an essential element in performance management systems because differentiated compensation encourages employees to achieve better performances and develop skills and competencies. The differentiated reward can be found within organizations in the form of three systems: the performance-related reward system, the competency-related reward system, and the contribution-related reward system. Performance-based payment ensures the relationship between performance and rewards [5]. Kirschner et al. [44] believe that, in healthcare, carrying out the activity and the constant contact with the patient are factors that allow the implementation of a performance-based reward program, in which financial rewards are at the heart of the reward system. Several actions affect the reward system, both from the point of view of the organization and the individual [45]. However, the reward is personal, and its vision differs from one individual to another [26]. However, managers must ensure that differentiated rewards are based on fairness and equity in evaluating performances to avoid conflict within the organization. This is why it is essential to assess the impact of equity on the reward package establishment and management and the two major components: financial motivation and recognition.

### 2.3. Factors Affecting Employee Performance and the Effect of Motivation on Organizational Performance

Employees’ motivation influences their performances [45,46]. Highly motivated employees are efficient and energetic, and produce high-quality results [47,48]. Most private organizations can achieve their goals through ensuring effectiveness and efficiency, which are achieved by motivating employees [16]. A good organizational performance is achieved due to the organization’s activities being carried out efficiently and effectively and the performance of employees during the organization’s mission. Wilton [25] stated that increased employee satisfaction and motivation reflect increased performance and organization results.

According to [49], the performances of organizations are the result of employee satisfaction. Therefore, the performance-based reward system best relates reward policies to labor productivity and employee performance [50]. Other differentiated reward systems (skill-based reward, contribution-based reward) can also positively influence labor productivity and employee performance if properly established [51]. Motivation has a significant role in many of the health workforce’s challenges, as employee performance and motivation are preconditions for organizational success. To increase employees’ motivation for vision and mission, each organization should raise staff living standards and provide a pleasant and performance-friendly organizational climate [52,53,54].

Employees’ motivation and good performances are indispensable in healthcare due to rivalry with the private sector. A necessary management activity is the motivation of human resources because the healthcare organizational managers find that they are competitors based on common sources of competitiveness [5]. As a result, organizational managers in healthcare need to develop effective strategies to motivate employees to improve performance and increase employee retention to achieve organizational objectives. Many healthcare workers may leave their companies due to a lack of motivation. Fried and Fottler [55] agreed that the turnover rate in healthcare institutions is an extensive issue. Employee retention could be enhanced through job satisfaction, recognition, social status, and reward [10]. Another vital method of employee retention is recognizing and appreciating employees’ healthcare efforts and social status [9]. Retaining healthcare professionals in public hospitals will help minimize the healthcare shortage.

Human resources for a healthcare organization are essential, so hospital managers should ensure that employees are satisfied with the job [55,56]. Niles [10] added that the salary of directly productive support staff (s, pharmacists, nurses) represent a significant factor that can affect employee satisfaction and retention because, for these positions, the benefits are inadequate, the high stress, the poor advancement opportunities and there are shortcomings in terms of recognition of merit and appreciation. The working environment of healthcare employees and organizational commitment can also induce employee retention [5,25,35,46]. In addition, employees’ perceptions of professional satisfaction may be relevant to employee retention.

Delery and Doty [57] consider that performance-based rewards are the strongest predictor of organizational performance. Merit-based promotion can be considered as a form of performance-based rewarding and can also be considered as an essential ingredient in organizational reward systems, encouraging retention and employees to perform better. Appropriate reward policies are also effective in lowering employee turnover. Baker [58] showed that effective reward plans were related to higher revenues, increased profits, and lower costs. Paul and Anantharaman [59] found that rewards directly influence performance in a similar study. To be fair, rewards practices and policies must be aligned with objectives.

Based on the theoretical considerations concerning the way in which the reward package is established, we formulated the third hypothesis of the research on the influence of rewards on human resource performances in healthcare:

**Hypothesis** **3** **(H3).** *The way the reward package is established and managed positively influences financial motivation and recognition (non-financial motivation) in ensuring a good self-perceived performance in employees*.

The way in which the salary package is structured (by correctly balancing financial and non-financial rewards, according to the perceived needs of employees) is a mediating factor in the relationship between financial motivation and recognition on the one hand and employees’ efforts to achieve the desired performance. In addition to the establishment pattern, the way in which this package is managed by ensuring fairness and equity in awarding rewards is also a key factor influencing financial rewards and recognition.

Issues such as rewards and performance are a chief concern for managers of hospital units from Romania [6]. The lack of motivational strategies and inadequate reward systems of employees at the level of hospital institutions in Romania negatively affects the organization’s performance [1,6]. Therefore, we believe that empirical studies are needed to highlight the factors that can better motivate employees and improve performance.

In Table 1, we present a summary of research issues with bibliographic sources.

## 3. Materials and Methods

**Figure 1:**
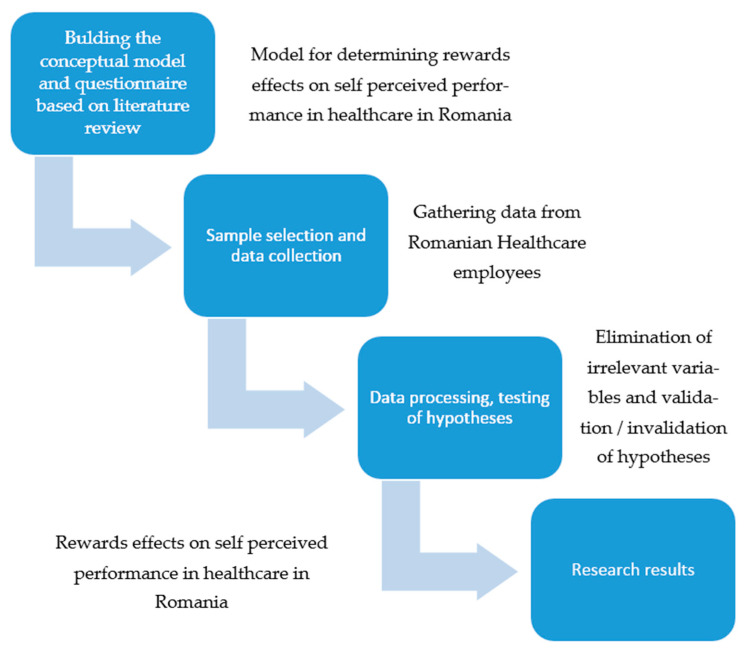
Research flowchart. Source: developed by authors.

Based on the theoretical approach of rewards in healthcare [8,9,10,11] and contingent and non-contingent rewards of Podsakoff et al. [60,61,62], this work aims to explore the effects of reward policies that could be implemented at the hospital level to improve employees’ performances. The research flowchart is shown in Figure 1.

The population selected for this case study includes emergency hospital staff from Romania. This study’s selected population represents both productive and non-productive employees (medical professionals, nurses, labor workers, and administrative staff). For the construction of the initial sample, we chose proportional stratified sampling. The final sample was based on the individuals who answered the self-administered questionnaire. Out of 983 emergency hospital employees who were sent the questionnaire, 288 employees responded. Of these, 280 were deemed valid, and the responders were diverse in terms of age, gender, and type of employment (directly and indirectly productive and non-productive employees). The data collection process included the administration of questionnaires for a period of four weeks. After selecting participants, the questionnaires served as the primary tool for data collection. To answer the main research question and ensure validity, employees answered a series of questions structured in a questionnaire consisting of closed multiple-choice questions (Table A1). The questionnaire was constructed based on the results of previous research. Table 1 contains the sources that formed the basis of the questionnaire.

Given that our research is based on self-report data, common method bias can have a negative effect on the validity of the results [63,64]. To combat this risk, we used cross-source data (the questionnaire was administered to rewarding leaders and subordinates who evaluate the effects of rewards on performance). Selection bias was combatted by compiling a representative sample for health workers in Romania, as can be seen in Table 1. Non-response bias was overcome by using various distribution methods (personal email, telephone, through representatives). To mitigate acquiescence bias, we did not use leading questions. Furthermore, we varied questions and answers in order to mitigate the answer primacy bias.

Table 2 presents the descriptive statistics characterizing the selected sample.

Within the sample, the proportion of female employees is higher, respecting the staff structure. The asymmetry coefficient is negative, indicating a right-angled distribution. By analyzing the statistical information obtained, we found that the average age of respondents is about halfway through the interval, around 43 years. The asymmetry coefficient is slightly positive, indicating a left-inclined distribution.

The structure of employees regarding education is balanced, slightly inclined to the left, illustrating that nurses and other categories of auxiliary staff represent a large part of the medical staff. A slightly negative asymmetry coefficient can be observed regarding the seniority in work, which indicates average seniority of around 17 years. In correlation with the average age, it can be stated that the average age of entry into the profession is 26 years (influenced by the duration of studies related to the initial training of medical staff). Seniority in the organization has values close to seniority, which indicates low employee mobility. For most of the employees, the organization was their first employer. From the direct observations, many healthcare professionals enrolled after completing their studies in emergency hospitals and did not leave the organization, even though they were employed in private healthcare units. Moreover, in this case, the coefficient of asymmetry is slightly negative.

Analysis of the distribution by departments indicates a pronounced asymmetry to the right, with the majority of those selected in the sample being employed in clinical wards. We selected only sixteen employees in the sample, respecting the proportion of management positions in the entire staff. This generates a strong inclination to the right of the distribution and a vaulting coefficient that indicates extreme values. For all other variables, distributions are platykurtic, with values dispersed over a more dispersed interval in the proximity of the mean.

According to the personnel category of descriptive statistics, the analysis shows a pronounced asymmetry to the right, resulting from the fact that a large part of the hospital staff is made up of auxiliary staff employed in different hospital departments. Furthermore, an unstable wage structure can be observed concerning net earnings, which is strongly influenced by the staff category, the length of service, and the section in which the employees are employed.

We applied structural equation modeling (SEM) to test the hypotheses’ validity, determining the mediation effects among the selected variables. Formula (1) is presented in the following [65,66,67]:

(1) $$\eta_{i}=\alpha_{\eta }+B\eta_{i}+\Gamma \xi_{i}+\zeta_{i}$$

η,ξ—endogenous and exogenous latent variables vectors, Β—matrix of the latent endogenous variables, Γ—matrix of the latent exogenous variables, ζ—disturbances, and i —cases.

We chose SEM to test the conceptual model of the research because SEM allows a more accurate determination of the influence relations that are established in a set of variables. SEM has the advantage of indicating the intensity of the relationships among latent variables and the constructs on which these variables depend—observable exogenous variables (items used in empirical research) and the meaning and intensity of relationships between latent (endogenous) variables. For our research, we chose PLS-SEM (partial least squares structural equation modeling) because we seek to identify “driver” constructs, Hair et al. [66] recommended this method for this purpose

**Figure 2:**
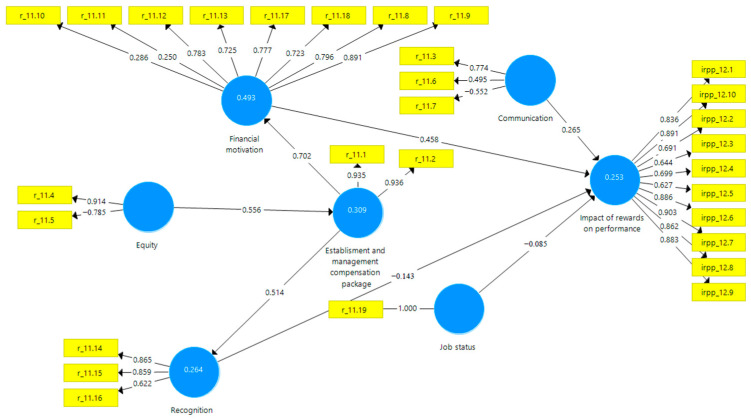
Conceptual model. Source: own construction developed using SmartPLS v.3.

The conceptual model of research of the influence of rewards on self-perceived performance in healthcare implemented through SmartPLS v.3 (SmartPLS GmbH, Bönningstedt Germany) is presented in Figure 2.

## 4. Results

**Figure 3:**
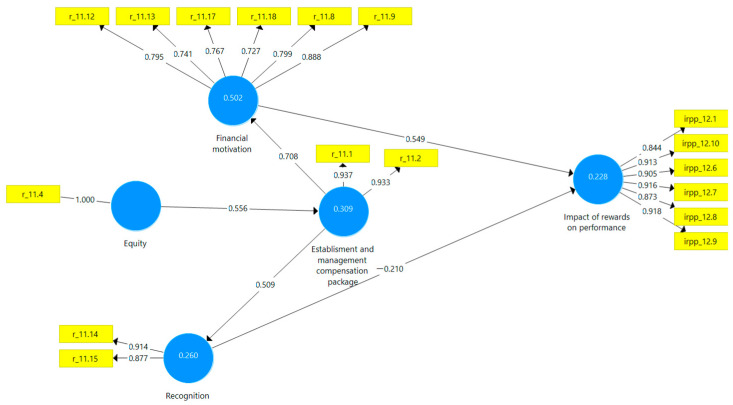
Modified model applied in healthcare. Source: own construction developed using SmartPLS v.3.

As can be seen from Figure 1, the proposed conceptual model was tested using PLS-SEM in the SmartPLS v.3 software. Items selected within the conceptual model (corresponding to observable external variables) were tested. Only items that met the validity requirement were retained (a load of each indicator for visible external variables has a value of over 0.7) (Figure 3).

The applied model shows good fit measures. The standardized root mean square residual (SRMR) is 0.75 (below 0.80), while the Normed Fit Index (NFI) is 0.913 (over 0.9). According to Hu and Bentler [68], a value of less than 0.08 is considered a good fit, and according to Lohmöller [69], NFI values above 0.9 usually represent an acceptable fit. The reliability and the validity (Table 3) are also good. Alpha Crombach and Reliability composite coefficients are above 0.8, and the average variance extracted (AVE) has values above 0.6 [54].

We applied the bootstrapping procedure using a two-tailed t-test (5% significance level) to test the hypotheses. Table 4 shows the path, significance level, and size effects.

The research on path coefficients (Figure 3 and Table 4) shows that financial motivation has a more significant influence than non-financial motivation (recognition) on the performance of human resources, which validates the H1 hypothesis. Furthermore, given the total effects (path coefficients) and the size effects (f2), it is evident that in the perception of the employees of the emergency hospitals selected in the study, the financial motivation has particular importance in the performances obtained.

To identify the importance of financial motivation according to the salary category, given the differentiated salary increases of healthcare workers in Romania, we analyzed the degree of association between the staff category and financial motivation. Following the analysis of the values recorded by the two variables (financial motivation and staff category), as a result of running the Chi-square test, we can say that there is a significant association between employees’ perceptions of financial motivation and the staff category (χ2 = 97,445, df = 12, *p* = 0.000 < 0.05). Table 5 shows the employees’ perceptions of financial motivation according to the staff category.

As can be seen from Table 5, for doctors, whose salaries have increased in the last three years, the financial motivation is no longer as important as in the case of the other categories of productive staff (nurses, pharmacists, laboratory staff) and non-productive staff (administrative and maintenance staff).

Therefore, it is necessary to structure the salary package fairly and ensure equity in its management. Following the research results, we noticed that ensuring equity positively impacts establishing and managing the reward package in general (total effects 0.556), which validates the H2 hypothesis. The indirect effects are exposed in Table 6.

The influences of equity on financial motivation are reduced (the indirect effects 0.394), given that there is a unitary salary scale established at the Ministry of Health for all employees in Romanian emergency hospitals.

To consolidate the results obtained in this research, we performed a univariate analysis of variance (ANOVA), defining equity, financial motivation, and recognition as independent variables and the establishment and management compensation package as the dependent variable.

(2) $$EMCP_{i}=\propto +Equity_{i}+Financial\;motivation_{i}+Recognition_{i}$$

Table 7 shows the parameters obtained based on the ANOVA model.

From the analysis of Table 7, we found, once again, that the H2 hypothesis is validated. Therefore, establishing and managing the reward package based on equity principles is necessary for the healthcare area. Although the financial and non-financial components of the reward package have equal relative influences, the equity with which they are distributed is much more critical in the perception of healthcare employees.

From the investigation of the influences established between the variables illustrated in Figure 3 and Table 4, the positive influences of the compensation package on the recognition (total effects 0.549) and financial motivation (total effects 0.708) are established and managed. To identify the influences of the way the compensation package is established and managed (EMCP), financial motivation, and recognition on employees’ self-perceived performances (IRPP), we used the analysis of artificial neural networks. The model used, multilayer perceptron (MLP), allows the evaluation of the influences of a set of variables in the input layer on another set of variables in the output layer through a hidden layer. In the case of our research, we defined input variables as EMCP, financial motivation and recognition, and output variables as IRPP. Employees’ perceptions of reward and performance are the hidden layer. The MLP model also implies the existence of biases in the form of external factors that act on the hidden layer (variables related to the individual characteristics of employees) and on the output layer (variables related to the reward system in the organization or similar organizations). The formula for this type of function is as follows (3):

(3) $$f\left(n\right)=\frac{1}{1+e^{-n}}=\frac{e^{n}}{e^{n}+1}$$

*n*—input variable; *f*(*n*)—output variable.

**Figure 4:**
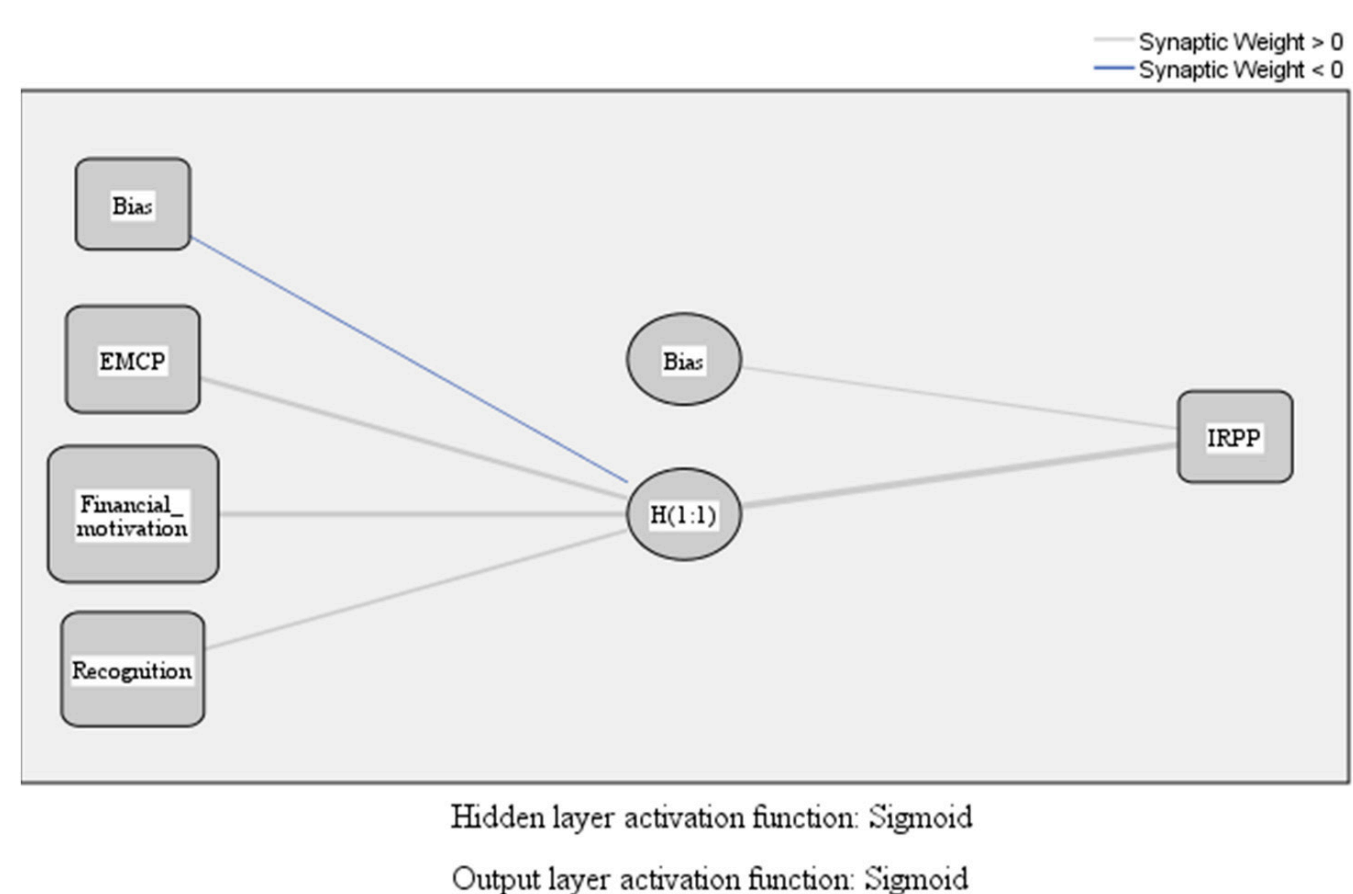
MLP model. Source: developed using SPSS v.20.

Figure 4 shows the relationships within the MLP model.

The relative error of the model in the test phase was 0.826. Table 8 shows the values for the predictors of the MLP model.

**Figure 5:**
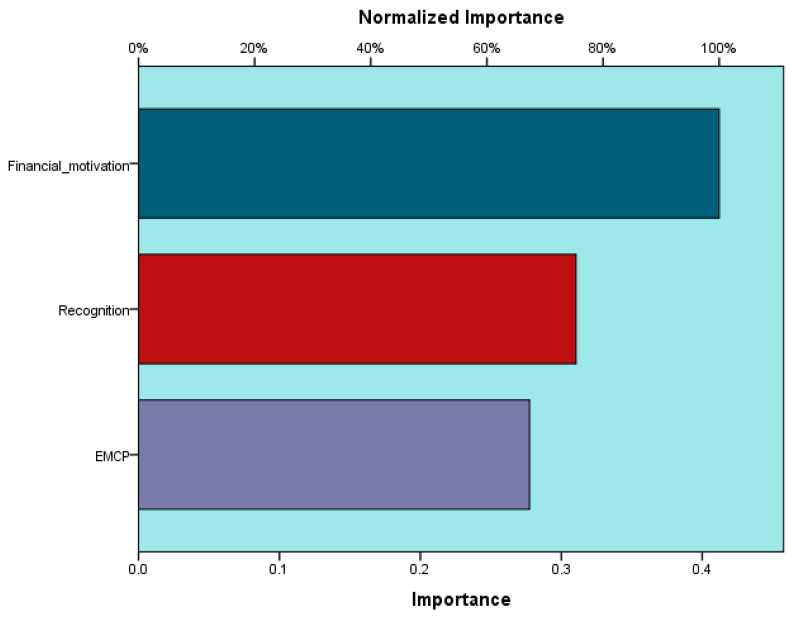
Importance of independent variables in the MLP model. Source: developed using SPSS v.20.

The analysis of Figure 4 and the data in Table 8 demonstrate the positive effects of how the compensation package is established and managed (EMCP), financial motivation, and recognition on employees’ self-perceived performances (IRPP) validates the H3 hypothesis. Figure 5 shows the absolute and normalized importance calculated for the input variables (EMCP, financial motivation, and recognition) in terms of the influence on the output variables (employees’ self-perceived performances) through the hidden layer (employees’ perceptions).

The validity of the H3 hypothesis is confirmed once again by studying the absolute and normalized importance of the input variables. The way the reward package is established and managed and an efficient allocation of financial and non-financial rewards are essential factors determining if employees achieve good individual and organizational performances.

## 5. Discussion

By researching the impact of reward policies on employees’ self-perceived performances, this paper aims to answer several questions: How can hospital employees be motivated to increase performance? What is the effect of the reward policy on employees’ self-perceived performance? Can the increase in the human resources level reward lead to improved medical efficiency indicators, given the precariousness of the non-human resources available to hospitals? This paper explores how reward policies improve human resources and organizational systems’ inputs, flows, and outcomes. By analyzing various aspects of employee motivations, including determinants, the mechanisms by which reward policies generate expected results, and how tangible rewards affect other medical staff motivations, this paper contributes to understanding the impact of reward policies on Romanian employees’ performance in healthcare.

The management of an organization must understand that performance is multidimensional, which highlights the many different aspects of behaviors that can influence the achievement of organizational goals. For example, [70] showed that for a better understanding of employee performance, the traits, behavioral approaches, and outcomes could be used to measure performance and implicitly establish reward levels. Verhulst and DeCenzo [32] stated that the motivational process ensures the link between the employee’s reward and his individual performance is known as performance or reward evaluation. Therefore, financial motivation and recognition are beneficial tools that management can use to improve motivation and performance [5]. Recognition can lessen perceived problems that medical employees working in disadvantaged areas may face because individuals want recognition for good work from the community in which they work [3,5,18,55]. Repaying employee accomplishments by rewarding or recognizing them gives the employee a sense of value and professional satisfaction. However, according to the results of our research, financial motivation has a much more significant influence on the performance of healthcare employees than the recognition, especially among the categories of non-productive auxiliary staff and directly productive staff, whose salaries have not increased as much as doctors’ salaries (H1 hypothesis). In emerging countries with lower average incomes, the motivating financial factor is decisive. Financial motivation is essential in healthcare, but when financial needs are satisfied at a certain level, non-financial motivation can effectively influence the employees’ efforts. The permanent recognition of merits and creating a favorable work environment become necessary landmarks to ensure an adequate motivational system aligned with organizational performance [29]. The reward is a measure of equity [45]. Employers prefer to pay employees low salaries to minimize costs, while employees prefer a maximum return for their efforts [5]. Yang [71] showed that fair pay is the most compelling reason to work for an organization.

Similarly, the results of our research show that ensuring equity has a positive impact on how the reward package is established and managed and, in particular, on financial motivation (H2 hypothesis). Conversely, a lack of equity, especially financial motivation, has a detrimental effect on employee performance. However, equity is viewed as a whole. It does not refer only to the financial reward since a unitary salary scale is established at the Ministry of Health for all employees of emergency hospitals in Romania. Therefore, to increase motivation, the reward package of healthcare employees must be structured and managed based on the principles of equity.

Although performance-based rewards can motivate employees, sometimes employees perceive it as a control mechanism and reward plans have the counter effect [72,73]. Management can establish motivational tools influencing employees’ behavior and motivation [5]. For example, during the economic crisis in Romania in 2008–2011, the private organization used financial incentives as the best motivator, while in public institutions, the reward system was in sharp decline [27]. However, in developed countries, financial reward occupies a lower position on the motivational scale because minimum wage levels are sufficient for a decent living [23]. Nevertheless, other authors showed that financial motivation remains the essential motivation strategy [11,13,71]. Similar to previous research, the results of our study (H3 hypothesis) show that the way the reward package is established and managed positively influences, primarily the financial motivation and recognition (non-financial motivation). The efforts to achieve desirable performance depend on how is structured and managed the reward package for healthcare employees and settled a balance, based on individual needs, between financial and non-financial motivation.

Over time, it has been found that there is a significant relationship between employees’ rewards and performance. Like [74], we consider an organizational ability to attract, motivate, and retain employees by offering competitive salaries and appropriate rewards to affect the performance and growth of organizations positively. In areas where employees work directly with clients, Ines and Pedro [75] found that the reward system significantly affects performance. Innovative reward strategies lead to increasing employees’ performance.

## 6. Conclusions

Following this research, we concluded that the effects of financial rewards on employees’ self-perceived performances are of paramount importance. Financial rewards are the most important incentives to increase performance, being motivational factors contributing substantially to meeting physiological and security needs. On the other hand, non-financial rewards are necessary to have a long-term motivational effect. The creative use of personalized non-financial rewards strengthens positive behaviors and improves employees’ performances. Employee involvement in decision making, job enrichment, job expansion, effective communication, organizational climate, well-structured training programs, good working environment, recognition, and performance feedback can meet the internal needs of individuals who are essential in motivating and increasing performance. This study showed that rewards impact employees’ self-perceived performances but have different effects, affecting performance.

### 6.1. Theoretical Implications

This paper aims to help reduce the gaps in the literature on reward policies in healthcare areas. We offer a multidimensional model for measuring the influence of rewards on employees’ self-perceived performances. The model allows for, by successive application, fine-tuning in establishing which methods of motivation are more useful at a given time to increase individual performances. The model also shows that the lack of equity in applying compensation means can lead to a drastic decrease in performance through the detrimental influence on financial motivation. Furthermore, this helpful tool can be used to carry out similar studies on other hospital institutions in Romania or elsewhere. Finally, the tool has the merit of grouping reward variables and allowing an external manager or consultant to analyze the impact of different reward strategies on employees’ self-perceived performances.

The conclusions of this paper contribute to a more effective healthcare practice based on the improvement of the reward system. The results could also give a perspective on developing a strategy to improve employees’ job performance. Improving employees’ self-perceived performances could help patients and ensure a lower consumption of financial resources from taxpayers.

### 6.2. Managerial Implications

Health managers can use the conclusions to increase the organizational commitment to improve the performance and the hospital unit image. In addition, this study’s findings could contribute to social change by providing knowledge to healthcare managers about the relevance of employees’ rewards and raising awareness of the variables that can influence employee productivity in the workplace. Finally, the model provides managers with a tool to determine what motivation is needed at a given time and the relationship between the reward package and employee performance. The main conclusions that can be drawn from the empirical study can be summarized as follows:

- Financial motivation is essential in healthcare, but when financial needs are met at a certain level, non-financial motivation can effectively influence the efforts of employees in order to achieve individual and organizational performances;
- To eliminate the sources of conflict and increase the motivation to achieve desirable performances, it is necessary that the reward package of healthcare employees be structured and managed based on the principles of equity;
- The efforts to achieve desirable performance depend on how is structured and managed the reward package for healthcare employees and settled a balance, based on individual needs, between financial and non-financial motivation.

The results of such studies can help hospital managers calibrate their reward systems, orient them towards performance, skills, or contributions, increase employees’ self-perceived performance, make the medical practice more efficient, and, ultimately, substantially improve patients’ health and their communities. In addition, improving employees’ performance could benefit patients by increasing the country’s health status, economic development, and economic growth by strengthening the national health system. Resource efficiency is essential for any organization [76] and especially for healthcare organizations. Along with eco-efficiency [77,78], the efficiency of human resource utilization is the pillar of the sustainable development of a country or region.

### 6.3. Limitations and Further Research

During the study, the responses collected from employees of several emergency hospitals in Romania in healthcare were analyzed, representing a geographical and cultural limitation. A limitation of the study deriving from the data collection method is the method of measuring the performance. This paper only considers self-perceived performance, not objectively measured performance. Therefore, the study results are not generalizable but can be applied to emerging countries with the same situation as Romania, where phenomena such as drain brain in healthcare occur. In further research, which may involve more human, financial, and institutional resources, the study could extend the area to several emergency hospitals in other countries to ensure data comparability. Additionally, in future studies, we will analyze the impact of reward, both on self-perceived performance (soft) and on measured performance (hard).

This study aims to clarify the vision of reward policies and their effects on self-perceived performance. Employees hold information about reward processes and may have a different view than health managers. The findings in this paper could help other healthcare managers raise their organizational performance, ensuring adequate motivation and employee retention. Future research focusing on health organizations could continue expanding this research line and building knowledge to help health managers manage performance.

## Figures and Tables

**Table 1:** Summary of research issues by bibliographic sources.

	General Rewards	Financial Motivation	Recognition	Equity
	Hunter, 2012 [24]	Chaudhry et al., 2011 [36]	Galletta et al., 2011 [46]	Henne and Locke, 1985 [40]
Concepts	Dickson et al., 2018 [30]	Baker, 2002 [58]	Fernet, 2013 [47]	Mondy, 2013 [39]
	Wilkinson, 2019 [45]	Pinto, 2011 [33]	Wilkinson, 2019 [45]	Torrington et al. 2017 [29]
	Wilton, 2019 [25]	Wilkinson, 2019 [45]	Wilton, 2019 [25]	Wilton, 2019 [25]
	Delery and Doty, 1996 [57]	Chaudhry et al., 2011 [36]	Paul and Anantharaman, 2003 [59]	Evan and Simmons (1969) [41]
Impact on performance	Choong et al., 2012 [37]	Judge et al., 2010 [49]	Lai and Chen, 2012 [19]	Ittner et al. (2003) [43]
	Yáñez-Araque et al., 2012 [53]	Visconti and Morea, 2020 [48]	Jankelová, 2021 [56]	Bao and Wu (2017) [42]

Source: Developed by the author based on bibliographic sources.

**Table 2:** Descriptive statistics of the selected sample.

	Min	Max	Mean	Standard Deviation	Skewness	Kurtosis
Gender	1	2	1.71	0.455	−0.970	−1.092
Age	2	5	3.50	0.881	0.131	−0.653
Education	1	5	2.57	1.001	0.423	−0.047
Work seniority	1	6	3.60	1.301	−0.063	−0.425
Organization seniority	1	6	3.54	1.369	−0.092	−0.555
Department	1	3	1.60	0.841	0.879	−1.004
Job	1	2	1.94	0.234	−3.900	13.597
Staff Category	1	4	2.97	1.142	−0.725	−0.927
Income	1	7	3.51	1.879	0.794	−0.945

Source: Developed by the author based on the collected data.

**Table 3:** Reliability and validity.

	Cronbach’s Alpha	rho_A	Composite Reliability	AVE
Equity	1	1	1	1
Establishment and management remuneration package	0.857	0.857	0.933	0.875
Financial motivation	0.877	0.88	0.907	0.621
Impact of rewards on performance	0.951	0.958	0.961	0.803
Recognition	0.755	0.769	0.89	0.802

Source: own construction developed using SmartPLS v.3.

**Table 4:** Path coefficients.

	Path Coefficients	T-Statistics	*p*-Values
Recognition −> Impact of rewards on performance (H1)	−0.210	0.825	0.410
Financial motivation −> Impact of rewards on performance (H1)	0.549	2.214	0.027
Establishment and management remuneration package −> Recognition (H3)	0.509	4.457	0.000
Establishment and management remuneration package −> Financial motivation (H3)	0.708	10.224	0.000
Equity −> Financial motivation (H2)	0.134	2.539	0.011
Equity −> Establishment and management remuneration package (H2)	0.556	5.053	0.000

Source: own construction developed using SmartPLS v.3.

**Table 5:** Employees’ perceptions of financial motivation according to the staff category.

		Staff Category				Total
		Auxiliary Staff	Laborants, Pharmacists	Nurses	Doctors	
1	Count	0	0	0	4	4
	% within staff category	0.0%	0.0%	0.0%	3.2%	1.4%
	% of Total	0.0%	0.0%	0.0%	1.4%	1.4%
2	Count	0	0	0	24	24
	% within staff category	0.0%	0.0%	0.0%	19.4%	8.6%
	% of Total	0.0%	0.0%	0.0%	8.6%	8.6%
3	Count	20	4	0	32	56
	% within staff category	38.5%	14.3%	0.0%	25.8%	20.0%
	% of Total	7.1%	1.4%	0.0%	11.4%	20.0%
4	Count	20	12	36	52	120
	% within staff category	38.5%	42.9%	47.4%	41.9%	42.9%
	% of Total	7.1%	4.3%	12.9%	18.6%	42.9%
5	Count	12	12	40	12	76
	% within staff category	23.1%	42.9%	52.6%	9.7%	27.1%
	% of Total	4.3%	4.3%	14.3%	4.3%	27.1%
Total	Count	52	28	76	124	280
	% within staff category	100.0%	100.0%	100.0%	100.0%	100.0%
	% of Total	18.6%	10.0%	27.1%	44.3%	100.0%

Source: own construction developed using SPSS v.20 (SmartPLS GmbH, Bönningstedt Germany).

**Table 6:** Indirect effects.

	Path Coefficients	T-Statistics	*p*-Values
Equity -> Financial motivation (H2)	0.394	4.425	0.000
Equity -> Impact of rewards on performance	0.156	2.464	0.014
Equity -> Recognition	0.283	3.172	0.002
Establishment and management compensation package -> Impact of rewards on performance	0.281	3.104	0.002

Source: own construction developed using SmartPLS v.3.

**Table 7:** Parameter estimates for ANOVA.

Parameter	B	Std. Error	t	Sig.	Partial Eta Squared
Intercept	−0.577	0.364	−1.584	0.114	0.009
Recognition	0.269	0.079	3.405	0.001	0.040
Equity	0.631	0.080	7.907	0.000	0.185
Financial motivation	0.286	0.089	3.212	0.001	0.036

Source: own construction developed using SPSS v.20.

**Table 8:** MLP model predictors.

Predictor		Predicted	
		Hidden Layer 1	Output Layer
		H(1:1)	IRPP
Input Layer	(Bias)	−0.222	
	EMCP	1.239	
	Financial motivation	1.523	
	Recognition	0.927	
Hidden Layer 1	(Bias)		0.737
	H(1:1)		1.731

Source: developed using SPSS v.20.

## Data Availability

Not applicable.

## Appendix A

**Table A1:** Items of the questionnaire on the perceptions of employees on reward policies and performance.

Major Issues	Questionnaire Items
Employee Reward Features	1. The salary package is well structured (r_11.1). 2. The salary package is well managed by the management of the unit (r_11.2). 3. Employees receive appropriate and timely notifications and information on any changes that affect their salary packages (r_11.3). 4. Rewarding employees ensures equal rewards for the same type of work (r_11.4). 5. My colleague from another department with the same qualification receives a higher salary income than me (r_11.5). 6. Employees are well informed and involved in formulating, discussing, and implementing rewards policies (r_11.6). 7. I do not know what happens in terms of pay in the organization where I work (r_11.7). 8. Salary income is satisfactory concerning the work they submit (r_11.8). 9. I feel motivated by the financial rewards I receive (r_11.9). 10. Increasing the financial reward will motivate me to improve my future performance (r_11.10). 11. Financial rewards positively affect the work environment and the organizational climate (r_11.11). 12. The salary rewards are nationally competitive (r_11.12). 13. The salary rewards are internationally competitive (r_11.13). 14. I feel appreciated for my work and my achievements (r_11.14). 15. The hospital recognizes my contribution to organizational success and efficiency (r_11.15). 16. I believe that my job is essential for the success and effectiveness of the hospital (r_11.16). 17. The structure of the reward system improves employee motivation and organizational efficiency (r_11.17). 18. The salary income I receive motivates me to contribute to the success and effectiveness of the organization (r_11.18). 19. The hospital where I work is better than private medical centers (r_11.19).
Effects of the appropriate reward policy on self-perceived performance	1. Motivate employees to work better (irpp_12.1). 2. Improves employee punctuality at work and reduces employee absenteeism (irpp_12.2). 3. Improves the desire of employees to make additional guards (irpp_12.3). 4. Increase employees’ commitment to the organization, and therefore employees work harder and better (irpp_12.4). 5. Increases employees’ willingness to learn new skills and practice these skills in the workplace (irpp_12.5). 6. Creates a healthy work environment and improves employee health (irpp_12.6). 7. Create a perfect working relationship between management and employees to increase performance (irpp_12.7). 8. It makes employees feel appreciated and give everything they can (irpp_12.8). 9. Attracts and motivates qualified staff to work better (irpp_12.9). 10. Reward the employees involved for high efforts and performance (irpp_12.10).
